# Severe Paediatric Asthma Collaborative in Europe: real-world data on children on biologics

**DOI:** 10.1183/23120541.00709-2024

**Published:** 2025-06-23

**Authors:** Norrice M. Liu, Mariëlle W. Pijnenburg, Antoine Deschildre, Ines de Mir-Messa, Sigve Adalen, Flore Amat, Luna Antonino, Priscille Biermé, Maynor Bravo-Lopez, Karin C.L. Carlsen, Silvia Carraro, Pierrick Cros, Celine Delestrain, Carolina Diaz Garcia, Ralph Epaud, Grazia Fenu, Valentina A. Ferraro, Louise Fleming, Laurence Hanssens, Anouk Heine, Géraldine Labouret, Maria-Chiara Leoni, Amelia Licari, Enrico Lombardi, Alejandro Lopez Neyra, Christophe Marguet, Julie Mazenq, Santiago Pérez Tarazona, Juan Carlos Ramos Díaz, Cyril Schweitzer, Elena Spada, José Valverde Molina, Stijn Verhulst, Stéphanie Wanin, Franca Rusconi

**Affiliations:** 1 Centre for Genomics and Child Health, Blizard Institute, Queen Mary University of London, UK; 2 Erasmus University Medical Centre, Rotterdam, The Netherlands; 3 Hôpital Jeanne de Flandre, CHU Lille, France; 4 Hospital Universitario Vall d'Hebron, Barcelona, Spain; 5 Division of Paediatric and Adolescent Medicine, Oslo University Hospital, Oslo, Norway; 6 Assistance Publique – Hôpitaux de Paris Robert Debré Hospital, Paris, France - INSERM 1018 - Centre de Recherche en Epidémiologie et Santé des Populations, Epidémiologie Respiratoire Intégrative, Villejuif, France; 7 Laboratory of Experimental Medicine and Pediatrics, Department of Pediatrics, Faculty of Medicine and Health Sciences, University of Antwerp – Edegem, Belgium; 8 Hôpital Femme Mère Enfant, Hospices Civils de Lyon, France; 9 Faculty of Medicine, University of Oslo, Norway; 10 Women's and Children's Health Department, University of Padova, Italy; 11 Department of Pediatrics, CHU Morvan, Brest, France; 12 Centre Hospitalier Intercommunal de Créteil; University Paris Est Créteil, INSERM, IMRB; Centre des Maladies Respiratoires Rares (RESPIRARE), Créteil, France; 13 Hospital Universitario Santa Lucía, Cartagena, Spain; 14 Meyer Children's Hospital IRCCS, Florence, Italy; 15 Department of Paediatric Respiratory Medicine, Imperial Healthcare NHS Trust and Royal Brompton Hospital, UK; 16 Hôpital Universitaire des Enfants Reine Fabiola, Bruxelles, Belgium; 17 The Centre Hospitalier Universitaire de Toulouse, France; 18 Pediatric Unit, Department of Clinical, Surgical, Diagnostic and Pediatric Science, University of Pavia, Italy; 19 Hospital Infantil Universitario Niño Jesús, Madrid, Spain; 20 University of Rouen Normandie, INSERM, CHU Rouen, Department of Pediatric Respiratory and Allergic Diseases, Rouen, France; 21 APHM - Pediatric pulmonology Department - Aix-Marseille University, INSERM, INRAE, Marseille, France; 22 Hospital Universitario La Fe, Valencia, Spain; 23 Hospital Universitario Carlos Haya, Malaga, Spain; 24 CHRU de Nancy, Pediatric Department, Hôpital d'Enfants, Vandooeuvre, France; 25 DeVAH UR3450, Physiology Department, Université de Lorraine, vandoeuvre, France; 26 Laboratorio Della Conoscenza Carlo Corchia, Firenze, Italy; 27 Department of Pediatrics, Antwerp University Hospital – Edegem, Belgium; 28 Department of Pediatric Allergology, Armand Trousseau University Hospital, Groupe Hospitalier Universitaire, AP-HP Sorbonne-University, Paris, France; 29 Department of Mother and Child Health, Azienda USL Toscana Nord Ovest, Pisa, Italy

## Abstract

**Introduction:**

Real-world data on children with severe asthma is scarce. We report characteristics of children with severe asthma already on biologics, enrolled in the Severe Paediatric Asthma Collaborative in Europe, a clinical research collaboration of the European Respiratory Society.

**Methods:**

We describe patient's characteristics including asthma control assessed with Global Initiative for Asthma (GINA) criteria, composite asthma severity index (CASI), exacerbations, unscheduled medical attendances, lung function and quality of life in children on biologic treatment because of severe asthma. We also assessed previous biologics use. Forced expiratory volume in 1 s, CASI, GINA, Paediatric Asthma Quality of Life Questionnaire score, exacerbations, unscheduled medical attendance and hospital admission comparisons in patients treated with different biologics were adjusted by age, sex and biologic therapy duration.

**Results:**

Among the 250 children (median age 13.2 years) recruited, 56.8% used omalizumab, 21.6% mepolizumab and 21.6% dupilumab. At enrolment, the dupilumab group was older (median 15.0 years), while the omalizumab group had been on biologic treatment the longest (median 622 days). Overall, 27% and 8% had partly controlled and uncontrolled asthma respectively, according to GINA. In the last 12 months, 52% and 29% had at least one and two exacerbations, respectively; airflow obstruction was found in 33%. 10% were admitted to hospital due to exacerbation. A previous switch from another biologic was recorded in 16%, predominantly due to nonresponse.

**Conclusions:**

Most children on biologics obtained good symptom control, but many still experienced asthma attacks. Switching between biologics was substantial. There is still an unmet need in severe paediatric asthma.

## Shareable abstract

**Analysis of the SPACE database showed most children with severe asthma treated with biologics have good symptom control, but many have switched between biologics and still have frequent asthma attacks. New biologics may have a role in these patients.** https://bit.ly/42heUZH

## Introduction

Severe asthma in children and adolescents only constitutes 2.1–4.5% of paediatric asthma [1]. Nevertheless, it poses large impacts on healthcare costs, asthma exacerbations and patients’ and families’ quality of life.

Biologic treatments have revolutionised severe asthma management. However, with much fewer children than adults affected by severe asthma [2], clinical trial and real-world descriptive data on paediatric patients’ characteristics and outcomes are limited [3–5].

Four monoclonal antibody biologic treatments are licensed in paediatrics by the European Medicines Agency: omalizumab, mepolizumab and dupilumab are licensed for age ≥6 years, and tezepelumab is licensed for age ≥12 years. In paediatrics, omalizumab can reduce asthma exacerbations and inhaled corticosteroid therapy [6]; mepolizumab can reduce exacerbations with no impact on other asthma outcomes [7]; and dupilumab has demonstrated reduced exacerbations and sustained lung function improvement [8, 9]. Efficacy data on the recently available tezepelumab are limited; the NAVIGATOR study only included 82 adolescents, where lung function, exacerbations and asthma symptoms were insignificantly different to controls [10, 11].

First-line choice and subsequent switching of biologics are restricted by national/local access and eligibility criteria, with decisions based on financial grounds and physicians’ experience, and varying clinical practice across countries, or even across centres within the same country [12–14]. No “real-world” information is available on the use of, or switching between, biologics in paediatrics.

The Severe Paediatric Asthma Collaborative in Europe (SPACE) is an expanding European network, supported by the European Respiratory Society (ERS) as a Clinical Research Collaborative, comprising 36 centres across 10 countries to date. This observational study reports the characteristics, including asthma outcomes, of a substantial paediatric severe asthma cohort treated with biologics, and reviews reasons for switching between biologics, identifying any unmet needs.

## Methods

The SPACE protocol and database design were previously described [15]. Briefly, children and adolescents aged 6–17 years, with a history of wheeze and spirometry evidence of asthma, whose asthma was managed at a specialised centre for ≥6 months, and were already on a biologic treatment, are included in this cross-sectional study between August 2021 and January 2024. Patients were diagnosed with severe asthma, defined as necessitating medium- to high-dose inhaled corticosteroids (ICSs) plus a second controller (Global Initiative for Asthma (GINA) [16] asthma treatment strategy steps 4 and 5), but remained uncontrolled (frequent exacerbations and/or chronic asthma symptoms) despite this therapy. The biologic choice was decided by patients’ physicians in individual participating centres, according to guidance from relevant authorities in each country.

Characteristics of patients who were already on biologics at enrolment in SPACE, including age, sex, body mass index (BMI), biologic therapy duration, respiratory treatments, aeroallergens sensitisation (positive skin prick tests and/or specific IgE), presence of atopic comorbidities, serum total IgE (kU·L^−1^) for those on biologics other than omalizumab, blood eosinophils (cells·µL^−1^) and fractional exhaled nitric oxide (*F*_ENO_) (ppb) were recorded.

Adherence was assessed using the test of the adherence to inhalers (TAI) score, which contains 10 items scoring from 1 (worst) to 5 (best), totalling 10 (worst adherence) to 50 (best adherence) [17].

The followings were also available at enrolment in SPACE: spirometry results (using prediction equations derived by the ERS Global Lung Initiative Spirometry Task Force [18]); the Composite Asthma Severity Index (CASI; comprising scores for symptoms and treatment in the last 2 weeks, exacerbations in the last 2 months, and lung function), where higher scores indicating worse severity [19]; GINA assessment questions on symptoms in the last 4 weeks [16]; quality of life using the Standardised Paediatric Asthma Quality of Life Questionnaire (PAQLQ; interviewer-administered, for all ages [20], where scores of ≥6 represent no/mild quality of life impairment and scores of <6 represent moderate/severe impairment [21]); severe asthma exacerbations (episodes requiring systemic corticosteroids for ≥3 days [22]), unscheduled medical attendances (emergency department or general practitioner/paediatrician) and hospital admissions because of asthma in the 12 months prior to enrolment. Previous biologics use and reasons to switch before enrolment were recorded and classified as lack of response, adverse event or uncontrolled comorbidity.

### Data collection

Data were recorded on web-based case report forms, held at the Health Informatics Centres, Dundee, UK. Chief investigators (NL, MP, AH and FR) can access anonymised data for database management through the “Safe Haven” platform, which allows data access and analysis but prevents data copying or alteration, ensuring complete data security. Data were reviewed regularly with feedback to site investigators to address inconsistencies and incompleteness. Data are protected according to the General Data Protection Regulation established by the European Union and national implementing legislation regarding research data. SPACE received ethical approval from the London Research Ethics Committee (UK) (reference 18/LO/0178), with local ethical approval for each participating centre. All parents/caregivers and patients ≥16 years gave informed consent.

### Statistical analysis

Categorical variables are described as number (percentage), and continuous variables as mean±sd or median (interquartile range; IQR) according to their distribution. The BMI at the time of spirometry testing was expressed as z-score using World Health Organization (WHO) BMI-for-age charts as a reference [23]. Spirometry measures were expressed as z-scores using the Global Lung Initiative as a reference [18].

Comparisons between treatment groups (omalizumab, mepolizumab and dupilumab) were tested using a Wilcoxon rank-sum test for variables with nonsymmetric distribution (age, duration of biologic treatment, TAI score, total IgE, eosinophils and *F*_ENO_), univariate linear regression for BMI z-scores, and generalised linear model with binomial distribution (sex, high-dose ICSs, long-acting β agonists (LABAs), sensitisation to aeroallergens, allergic rhinitis or conjunctivitis and atopic dermatitis). forced expiratory volume in 1 s (FEV_1_) z-score, CASI, GINA criteria, PAQLQ score, exacerbations (at least one or two systemic steroid courses), at least one unscheduled medical attendance and at least one hospital admission in the last 12 months were compared using appropriate generalised linear models adjusting for potential confounders (age, sex and duration of biologic therapy), which were selected due to their possible influence on these variables.

Specifically, the analysis of FEV_1_ z-scores and CASI scores were performed using linear regression; asthma control according to GINA criteria with generalised logit functions; PAQLQ scores using general linear model with gamma distribution and log link; exacerbations, unscheduled medical attendances and hospital admissions with logistic regression. Overall p-values were used to summarise comparisons across the treatment groups. The Benjamini–Hochberg method was used to control the false discovery rate at 0.05.

In a sensitivity analysis, we excluded patients who were treated with biologics for <16 weeks, which was deemed too short a duration to describe treatment outcomes such as FEV_1_, asthma control based on GINA, CASI and PAQLQ scores, exacerbations, hospital admissions and unscheduled medical attendances. An additional sensitivity analysis, excluding those treated with biologics for <12 months, was performed for exacerbations, unscheduled medical attendances and hospital admissions in the last 12 months.

SAS, v.9.4 (SAS Institute, North Carolina, USA), was used to process data and fit statistical models.

## Results

A total of 250 children and adolescents with a median age of 13.2 years were included, of which 97 (38.8%) were female and 22 were from publicly funded centres in six European countries (The Netherlands, Italy, Spain, Norway, Belgium and France). Their characteristics at enrolment are shown overall and are stratified by biologic treatments (table 1).

**TABLE 1:** Children and adolescents’ characteristics at enrolment into SPACE, as a group on a whole and subcategorised based on biologic therapy

	n=250 (unless specified otherwise)	Biologic			Comparison between biologics (p-value)			
		Omalizumab n=142	Mepolizumab n=54	Dupilumab n=54	Oma *versus* Mepo	Oma *versus* Dupi	Mepo *versus* Dupi	Overall
**Age, years**	13.2 (10.6–15.2)	12.9 (10.5–14.8)	12.9 (9.0–15.0)	15.0 (12.5–16.3)	0.98	**<0.001**	**<0.01**	0.0003^+^
**Females**	97 (38.8)	50 (35.2)	20 (37.0)	27 (50.0)	0.81	0.06	0.17	0.1629
**BMI z-score mean±sd**	0.67±1.5	0.67±1.5	0.94±1.6	0.39±1.3	0.26	0.23	0.053	0.16
**Duration of treatment, days**	483.5 (211–927)	622 (240–1134)	390 (205–683)	336 (176–665)	0.13	**<0.01**	0.45	0.0032^+^
**High-dose ICS^#^**	89 (35.6)	56 (39.4)	18 (33.3)	15 (27.8)	0.42	0.11	0.53	0.29
**LABA use**	226 (90.4)	126 (88.7)	51 (94.4)	49 (90.7)	0.16	0.67	0.46	0.48
**TAI score**	48 (44–50)	48 (44–50)	48.5 (44.5–50)	48 (43–50)	0.81	0.81	0.46	0.55
**Total IgE IU·mL^−1¶^**	n=180 604 (202–1351.5)	n=80 612 (283–1231.5)	n=48 403 (99.45–1407.5)	n=52 658.5 (193–1356)	0.24	0.99	0.54	0.29
**Eosinophils cells·µL^−1¶^**	n=227 366 (190–640)	n=125 400 (330–640)	n=52 200 (100–620)	n=50 390 (250–700)	**<0.01**	0.99	**0.036**	0.0045^+^
**Aeroallergens sensitisation^¶^**	n=245 218 (89.0)	n=140 137 (97.9)	n=51 34 (66.7)	n=54 47 (87.0)	**<0.001**	**0.02**	**0.01**	<0.0001^+^
**Allergic rhinitis or conjunctivitis**	n=249 198 (78.7)	n=142 125 (88.0)	n=54 27 (50.0)	n=53 44 (83.0)	**<0.001**	0.39	**<0.001**	<0.0001^+^
**Atopic dermatitis**	n=244 115 (47.1)	n=139 61 (43.9)	n=54 21 (38.9)	n=51 33 (64.7)	0.52	**<0.01**	**<0.01**	0.015^+^
***F*_ENO_ ppb**	n=133 17 (10.8–33)	n=68 17 (9.6–31)	n=26 27.5 (8–41)	n=39 15 (11–30)	0.70	0.92	0.51	0.55
**FEV_1_, z-score**	−0.67±1.29	−0.60±1.30	−0.49±1.40	−1.02±1.12	0.64^§^	0.19^§^	0.12^§^	0.28
**CASI**	5 (4–7)	5 (4–7)	5 (5–7)	5 (4–7)	0.67^§^	0.54^§^	0.85^§^	0.80
**GINA scores**								
Controlled	162 (64.8)	91 (64.1)	38 (70.4)	33 (61.1)	0.20^§^	0.38^§^	0.46^§^	0.30
Partly controlled	67 (26.8)	34 (23.9)	15 (27.8)	18 (33.3)				
Uncontrolled	21 (8.4)	17 (12.0)	1 (1.9)	3 (5.6)				
PAQLQ score	n=245 6.3 (5.6–6.8)	n=137 6.3 (5.6–6.8)	n=54 6.4 (5.9–6.8)	n=54 6.4 (5.5–6.9)	0.48^§^	0.74^§^	0.78^§^	0.77
**Exacerbations**								
≥2	72 (28.8)	42 (29.6)	15 (27.8)	15 (27.8)	0.67^§^	0.95^§^	0.77^§^	0.91
≥1	129 (51.6)	73 (51.4)	30 (55.6)	26 (48.1)	0.83^§^	0.59^§^	0.51^§^	0.80
≥1 unscheduled medical attendance	38 (15.2)	19 (13.4)	9 (16.7)	10 (18.5)	0.11^§^	0.88^§^	0.26^§^	0.26
≥1 hospital admission	24 (9.6)	10 (7.0)	10 (18.5)	4 (7.4)	0.045^§^	0.90^§^	0.11^§^	0.09

Data are presented as n (%), mean±sd or median (IQR). SPACE: Severe Paediatric Asthma Collaborative in Europe; Oma: omalizumab; Mepo: mepolizumab; Dupi: dupilumab; BMI: body mass index; CASI: Composite Asthma Severity Index; ED: emergency department; *F*_ENO_: fractional exhaled nitric oxide; FEV_1_: forced expiratory volume in 1 s; GINA: Global Initiative for Asthma; ICS: inhaled corticosteroid; IgE: immunoglobulin E; IQR: interquartile range; LABA: long-acting β agonist; PAQLQ: Paediatric Asthma Quality of Life Questionnaire; TAI: Test of the Adherence to Inhalers. Overall: p-value of the overall comparisons. ^#^: High dose as defined by GINA according to age groups (6–11 years and ≥12 years) [16]. ^¶^: Latest results available at recruitment to SPACE, tests might have been performed while on biologic treatments. ^+^: p-value less than the critical value according to the Benjamini–Hochberg procedure. ^§^: comparisons are corrected by age, sex and duration of therapy. Values in bold are significant comparisons after the Benjamini–Hochberg procedure.

Of the 250 patients, 142 (56.8%) were treated with omalizumab, 54 (21.6%) with mepolizumab and 54 (21.6%) with dupilumab. Biologics were used off-label in 13 (5.2%) patients, of which 5 (38.5%) were outside the eligible age range, 5 (38.5%) did not fulfil treatment eligibility criteria and 3 (23.0%) had unrecorded reasons. Most patients (81.2%, no difference in the three treatment groups) were white. The dupilumab group was significantly older than the omalizumab (p<0.001) and mepolizumab (p<0.01) groups, whereas no difference emerged between omalizumab and mepolizumab. Omalizumab therapy duration was longer with a median length of 622 days *versus* 390 days for mepolizumab and 336 days for dupilumab; significantly different between omalizumab and dupilumab only (p<0.01).

Despite biologics use, 89 (35.6%) patients remained on high-dose ICS, as defined by GINA [16] according to age, with no difference between groups; and 226 (90.4%) were using LABAs. Inhaled therapies adherence was intermediate: median (IQR) TAI score of 48 (44–50), very similar across groups. Regarding atopy, 89% of the patients were aeroallergens sensitised; sensitisation frequency was significantly lower in mepolizumab *versus* dupilumab (p=0.01) and omalizumab (p<0.001) groups, and higher in omalizumab *versus* dupilumab group (p=0.02). In all, 78.7% patients had allergic rhinitis/conjunctivitis with a lower prevalence in mepolizumab compared with omalizumab and dupilumab groups (p<0.001 for both comparisons); while 47.1% of the patients had atopic dermatitis with a higher prevalence in dupilumab than mepolizumab and omalizumab groups (p<0.01 for both comparisons). Median serum eosinophils were 366 cells·µL^−1^, with the mepolizumab group lower (200 cells·µL^−1^) than omalizumab (390 cells·µL^−1^; p<0.01) and dupilumab (400 cells·µL^−1^; p<0.01) groups; 59.6% and 48.1% of the mepolizumab and dupilumab groups had serum eosinophils measured while being treated with biologics. The median *F*_ENO_ was 17.00 ppb, with no difference between groups.

No significant difference between groups in FEV_1_ z-score or CASI score was observed. 64 patients (25.6%) had FEV_1_ z-scores of <−1.64 and 81 (32.4%) had FEV_1_/FVC z-scores of <−1.64. A substantial percentage of patients (64.8%) had controlled asthma according to GINA. Nevertheless, 67 patients (26.8%) had only partly controlled asthma and 21 (8.4%) had uncontrolled asthma, with no significant difference between groups. Median (IQR) PAQLQ in the total sample was 6.3 (5.6–6.8), indicating no/mild impairment in quality of life, with no difference between groups. In the previous 12 months, a substantial number of patients (72, 28.8%) had ≥2 exacerbations, 38 (15.2%) required ≥1 unscheduled medical attendance and 24 (9.6%) required ≥1 hospital admission due to asthma. Comparisons of FEV_1_, CASI, GINA, PAQLQ, exacerbations, unscheduled medical attendance and hospital admissions are also shown in supplementary figures S1–S8.

Of note, among the 162 patients with controlled asthma according to GINA, only 45 (27.8%) had no exacerbations or unscheduled medical attendances in the last 12 months, with PAQLQ >6 and FEV_1_ z-score of >−1.64.

Sensitivity analysis, in which patients treated with biologics for <16 weeks (n=14 in omalizumab, n=1 in mepolizumab and n=4 in dupilumab) were removed, confirmed the results of the main analysis (supplementary figures S9–S16). Similar results were obtained in the sensitivity analysis removing those who had used biologics for <12 months (n=51 in omalizumab, n=23 in mepolizumab and n=29 in dupilumab; supplementary figures S17–S20).

Of the 250 patients, 40 (16%) had switched from another biologic before enrolling into SPACE. In particular, 25 (62.5%) switched due to nonresponsiveness to the previous biologic. Detailed reasons for switching are shown in table 2. Following a biologic switch in these 40 patients, 22 (55.0%) obtained well-controlled asthma according to GINA; 13 (32.5%) only achieved partly controlled asthma with 10 of them having switched due to nonresponse to the original biologic; and 5 (12.5%) remained with uncontrolled asthma with 3 of them having had nonresponse to the previous biologic.

**TABLE 2:** Number of patients who had switched between different biologics by the time they were enrolled into SPACE, with reasons for switching

**Omalizumab (n=142)**	n=3 (2%) used mepolizumab before: 3: nonresponders to mepolizumab
**Mepolizumab (n=54)**	n=10 (18.5%) used omalizumab before: 7: nonresponders to omalizumab 1: started on immunotherapy and asthma control became poor 1: good asthma control but poor effect on severe vernal keratoconjunctivitis on omalizumab 1: finance reasons (private insurance)
**Dupilumab (n=54)**	n=19 (35.2%) used omalizumab before: 10: nonresponders to omalizumab 1: efficacy disappeared after 3 years 5: ongoing atopic dermatitis 2: allergy/anaphylaxis 1: side-effect (painful injection sites) with moderate response only n=6 (11.1%) used mepolizumab before: 3: nonresponders to mepolizumab; 1: severe atopic dermatitis 1: allergy/anaphylaxis 1: changed when dupilumab became available for patient's age. n=2 (3.7%) used omalizumab followed by mepolizumab before: 2: nonresponders to both omalizumab and mepolizumab

SPACE: Severe Paediatric Asthma Collaborative in Europe.

Of the 88 (35.2%) patients with partly or uncontrolled asthma at inclusion, 19 (21.6%) had already switched from another biologic, two of whom had been treated with all three biologics at different time points.

## Discussion

This is the largest European real-world dataset of severe paediatric asthma, describing clinical characteristics in children on three different biologics.

Most patients were adolescents. Almost all used a combination ICS–LABA inhaler, with >35% requiring high-dose ICSs, despite most having used a biologic for >6 months (median duration of >1 year). Most patients treated with omalizumab and dupilumab were sensitised to aeroallergens with allergic comorbidities, whereas these prevalences were lower in the mepolizumab group.

Reassuringly, good asthma symptom control was obtained in two-thirds and most had good quality of life. Although the mean FEV_1_ z-scores for all groups were within the normal cut-off of >−1.64 [24], there remained a quarter of patients with FEV_1_ z-scores of <−1.64, and a third with FEV_1_/FVC z-scores of <−1.64, representing significant airflow obstruction.

One of the several interesting observations in this study is that patients using dupilumab were older. We speculate this is because dupilumab is the newest amongst the three biologics, its licence was the last to lower from age ≥12 years to ≥6 years. Furthermore, the dupilumab cohort most likely started their biologic journey with an “older” biologic. Indeed, 27 of 54 (50%) dupilumab users in SPACE had switched from one or both other biologics (table 2), with none switching from dupilumab to another biologic. Statistical analysis was therefore adjusted for age as a potential confounder. Second, there were nonsignificantly proportionately more females using dupilumab. We hypothesise this is because the higher incidence of childhood asthma in males than females is reversed during puberty, with female sex hormones associated with delayed onset of bronchial hyperreactivity, poor asthma control and less remission [25, 26]. This relates to the observation of the dupilumab group being older, hence having proportionately more females. Sex was therefore treated as another potential confounder. Third, the mean BMI z-score was nonsignificantly higher in the mepolizumab group. Together with the observation of this group being less atopic, we speculate whether those treated with mepolizumab had different T2 phenotypes. Commonly, patients with allergic sensitisation or allergic comorbidities are treated with omalizumab and dupilumab, as illustrated by the higher prevalence of atopic dermatitis in our dupilumab cohort. Finally,16% of our cohort had trialled a different biologic before enrolment into SPACE, the majority switched due to nonresponse to the original biologic, with two of them still not achieving good asthma control despite trialling all three biologics.

Within the SPACE cohort, 64.8%, 26.8% and 8.4% achieved good, partly controlled and uncontrolled asthma according to GINA, respectively, similar to previous real-life studies: a paediatric French survey demonstrated 67%, 25% and 8% had good, partial and poor control at 52 weeks while on omalizumab [27]. Regarding exacerbations, for omalizumab, Busse *et al.* [28] found that 30.3% of patients (6–20 years) had ≥1 exacerbations in a 60-week placebo-controlled, double-blind trial, while Nakamura *et al.* [29] found that 15.1% of their first-time omalizumab patients (6–15 years) had ≥2 exacerbations in their 104-week surveillance. In our omalizumab cohort, 51.4% and 29.6% had ≥1 and ≥2 exacerbations requiring systemic steroids in the last 12 months, respectively. For mepolizumab, Gupta *et al.* [30] demonstrated that 47% of children (6–11 years) had ≥1 exacerbation in a 52-week, open-label, repeat-dose extension study, whereas 55.6% of our cohort had ≥1 exacerbation. For dupilumab, Bacharier *et al.* [9] showed that 22.9% of children (6–11 years) had ≥1 exacerbation in their 52-week placebo-controlled, double-blind trial, and 48.1% of our cohort had ≥1 exacerbation. Differences between our findings and previous literature are likely due to study conditions including follow-up, age and treatment durations. Additionally, in clinical trials, patients are vigorously followed-up, resulting in better adherence to all therapies, and better asthma control in placebo and treatment groups, whereas this cross-sectional analysis provides a snapshot of patients’ characteristics in a real-world setting.

This work's strengths include its pan-European nature, encompassing different practices across countries, collating a high number of patients with this rare condition. SPACE collects data on all variables recommended by the ‘Core Outcome Measure in Severe Asthma’ initiative [31], with the exception of ACT/cACT, which are presently added to the latest SPACE database. With our network of experts, SPACE seeks to generate consensus and guidance on biologic therapy continuation, discontinuation and switching; and to support clinicians’ decisions on biologic treatment strategies. SPACE follow-up data on a substantial cohort of well-characterised paediatric patients will facilitate research, clinical trials and quality-improvement initiatives.

There are limitations in this work. Although we aimed to identify unmet needs in patients who are on biologics, as a cross-sectional analysis, changes in characteristics, before and since starting on biologics cannot be assessed. Atopy and eosinophil data may be affected by biologics use and therefore are not very informative; mepolizumab is an eosinophil cytokine blocker and dupilumab can transiently increase serum eosinophils [32]. Longitudinal data will improve understanding on biological and clinical responses to biologics, especially in those requiring biologics switching; data on patients initiating on biologics and subsequent follow-ups are currently being collected within SPACE.

This study provided cross-sectional data on a large cohort of children with severe asthma treated with a biologic. Many questions on biologic treatment in these children remain unanswered. It is important to identify whether remission is achievable in children, and to determine its definition, taking into account paediatric specificities. Biologic therapies combination to cover different aetiologies is considered in adults but not described in paediatrics. Last, for those who have achieved good asthma control with biologics, the challenge lies in lack of long-term management strategies. It remains unclear if/when biologics can be discontinued, and how other asthma treatments should be adjusted during the weaning/discontinuation phase.

In conclusion, this observational study in children on biologic treatment because of severe asthma showed that the majority obtains good symptom control and quality of life and have normal lung function. However, a substantial proportion of these children remains partly or uncontrolled, with exacerbations, and switching between biologics is common. Prospective studies are needed to assess treatment response in individual children and compare this response between different biologics.
